# The impact of legal expertise on moral decision-making biases

**DOI:** 10.1057/s41599-020-00595-8

**Published:** 2020-09-23

**Authors:** Sandra Baez, Michel Patiño-Sáenz, Jorge Martínez-Cotrina, Diego Mauricio Aponte, Juan Carlos Caicedo, Hernando Santamaría-García, Daniel Pastor, María Luz González-Gadea, Martín Haissiner, Adolfo M. García, Agustín Ibáñez

**Affiliations:** 1 Departamento de Psicología, Universidad de los Andes, Bogotá, Colombia; 2 Departamento de Ingeniería de Sistemas y Computación, Universidad de los Andes, Bogotá, Colombia; 3 Centro de Investigaciones sobre Dinámica Social (CIDS), Salud, Conocimiento Médico y Sociedad, Facultad de Ciencias Sociales y Humanas, Universidad Externado de Colombia, Bogotá, Colombia; 4 Intellectus Memory and Cognition Center, Hospital Universitario San Ignacio, Bogotá, Colombia; 5 Departments of Physiology, Psychiatry and Aging Institute, Pontificia Universidad Javeriana, Bogotá, Colombia; 6 Instituto de Neurociencias y Derecho, INECO Foundation, Buenos Aires, Argentina; 7 Facultad de Derecho, Universidad de Buenos Aires, Buenos Aires, Argentina; 8 Neuroscience Laboratory, Torcuato di Tella University, Buenos Aires, Argentina; 9 Universidad de San Andrés, Buenos Aires, Argentina; 10 National Scientific and Technical Research Council (CONICET), Buenos Aires, Argentina; 11 Yale law School, New Haven, CT USA; 12 Faculty of Education, National University of Cuyo (UNCuyo), Buenos Aires, Argentina; 13 Global Brain Health Institute (GBHI), University of California San Francisco (UCSF), San Francisco, USA; 14 Universidad Autónoma del Caribe, Bogotá, Colombia; 15 Center for Social and Cognitive Neuroscience (CSCN), School of Psychology, Santiago de Chile, Chile

**Keywords:** Psychology, Science, technology and society

## Abstract

Traditional and mainstream legal frameworks conceive law primarily as a purely rational practice, free from affect or intuition. However, substantial evidence indicates that human decision-making depends upon diverse biases. We explored the manifestation of these biases through comparisons among 45 criminal judges, 60 criminal attorneys, and 64 controls. We examined whether these groups’ decision-making patterns were influenced by (a) the information on the transgressor’s mental state, (b) the use of gruesome language in harm descriptions, and (c) ongoing physiological states. Judges and attorneys were similar to controls in that they overestimated the damage caused by intentional harm relative to accidental harm. However, judges and attorneys were less biased towards punishments and harm severity ratings to accidental harms. Similarly, they were less influenced in their decisions by either language manipulations or physiological arousal. Our findings suggest that specific expertise developed in legal settings can attenuate some pervasive biases in moral decision processes.

## Introduction

In legal settings, decision-making ideally requires unbiased, rational, and shared good reasons to guarantee fair processes. Although traditional legal ethos conceives law primarily as a rational field in which affect or intuition must take a secondary place (Gewirtz, 1996), human decision-making is influenced by cognitive and emotional factors (Ames and Fiske, 2013; Greene and Haidt, 2002; Treadway et al., 2014). For instance, decisions about punishment of harmful third-party actions are frequently driven by emotional biases (Bright and Goodman-Delahunty, 2006; Buckholtz et al., 2008; Goldberg et al., 1999; Treadway et al., 2014). Likewise, people overestimate the damage caused by intentional harms compared to identical accidental harms, assigning more punishment and moral condemnation to the former (Ames and Fiske, 2013, 2015; Baez et al., 2014, 2016). Undeniably, the law sometimes expressly recognizes the influence of such elements (Guthrie et al., 2001). However, unlike rules and principles, non-rational considerations tend to be hidden or overlooked. Due to their relevance for legal contexts, our aim is to further illuminate the interaction of these factors in legal decision-makers. We explored the moral decisions of criminal judges, criminal attorneys, and controls, focusing on moral evaluation, punishment assignment, and harm assessment of third-party aggressions (Treadway et al., 2014). We evaluated the influence of (a) information on the transgressor’s mental state, (b) the use of gruesome language (GL) in harm descriptions, and (c) ongoing physiological states.

The three factors above are critical for decision-making. First, inferences of other mental states are a critical driver of moral (Baez et al., 2017; Guglielmo, 2015; Yoder and Decety, 2014) and legal (Buckholtz and Faigman, 2014; Greely, 2011) deliberations. Specifically, intentional harms, compared to identical accidental harms, are punished more severely, deemed morally worse, and judged to induce greater damage (Alter et al., 2007; Cushman, 2008; Darley and Pittman, 2003; Koster-Hale et al., 2013; Young et al., 2010, 2007). This biasing effect of intentionality on harm quantification persists even in the face of economic incentives to be objective (Ames and Fiske, 2013). In legal contexts, blameworthiness is judged, among other factors, by the mental state that accompanies a wrongful action (Buckholtz and Faigman, 2014). Also, punishment determinations require inferences about the beliefs, intentions, and motivations of the potential perpetrator (Buckholtz and Marois, 2012). Second, emotionally arousing elements, such as the use of gruesome language (GL) to describe harm, can bias decision-making. GL leads to significantly greater emotional responses (e.g., stress, anguish, shock) (Nuñez et al., 2016) which promote harsher punishments and boost the activity of the amygdala (Treadway et al., 2014), a key brain region for emotional processing and harm encoding (Bright and Goodman-Delahunty, 2006; Hesse et al., 2016; Salerno and Peter-Hagene, 2013; Shenhav and Greene, 2014). Effects of emotionally arousing elements have been reported even in legal contexts (Bright and Goodman-Delahunty, 2006). Gruesome evidence (e.g., autopsy images of severe injuries) typically provokes anger or disgust (Bright and Goodman-Delahunty, 2006; Salerno and Peter-Hagene, 2013; Treadway et al., 2014) and can influence mock-jurors’ verdicts of defendants’ guilt or punishment (Bright and Goodman-Delahunty, 2006, 2011; Whalen and Blanchard, 1982). However, the effect of such elements on decision-making has not been assessed in legal experts. Third, emotional responses at large are driven by ongoing physiological states, which also shape decision-making processes (Greifeneder et al., 2011; Lerner and Keltner, 2000; Winkielman et al., 2007).

Perhaps unsurprisingly, legal decision makers are not fully immune to implicit biases—unconscious, automatic responses that shape behavior (Greenwald and Banaji, 1995). Although judges’ deliberations have been proposed to hinge on facts, evidence, and highly constrained legal criteria (Guthrie et al., 2008), legal decisions may be affected by various biases. Numeric anchors influence how legal decision makers determine appropriate damage awards and criminal sentences (Englich et al., 2006; Rachlinski et al., 2015). For instance, legal experts (judges and prosecutors) have been observed to anchor their sentences on particular random influences (e.g., random numbers or prior criminal sentences in unrelated cases) (Englich et al., 2006). Judges’ decisions are also influenced by common cognitive illusions, such as framing (different treatment of economically equivalent gains and losses) and egocentric biases (overestimations of one’s own abilities) (Guthrie et al., 2001). Moreover, they may operate on implicit racial biases, which can affect their decisions (Rachlinski et al., 2008). White judges display an automatic preference for White over Black. Black judges carry a more diverse array of implicit biases: some exhibit a White preference, others exhibit no preference, and still others exhibit a Black preference. Moreover, these implicit associations influence judges’ decisions when the race of the defendant is subliminally manipulated. After exposure to a Black subliminal prime, judges with strong White preferences make harsher judgments of defendants, while judges with strong Black preferences are more lenient. However, judges are able to monitor and suppress their own racial biases when consciously motivated to do so (Rachlinski et al., 2008). In addition, although experimental research is lacking, preliminary evidence suggests that emotions and reactions to litigants may influence judges’ decisions (Wistrich et al., 2015). Specifically, affect influences judges’ interpretation of the law, biasing decisions in favor of litigants who generate positive affective responses (Wistrich et al., 2015).

As a fundamental component of human culture, morality involves prescriptive norms regarding how people should treat one another, including concepts such as justice, fairness, and rights (Yoder and Decety, 2014). In addition to ordinary moral norms, the law exerts a major regulatory role in social life (Schleim et al., 2011). Indeed, moral and legal decision-making have been linked to broadly similar neural correlates, suggesting a considerable overlap in their underlying cognitive processes (Schleim et al., 2011). Both moral and legal judgments recruit a brain network including the dorsomedial prefrontal cortex, the posterior cingulate gyrus, the precuneus, and the left temporo-parietal-junction (Schleim et al., 2011). These regions are typically active when thinking about the beliefs and intentions of others (Saxe and Kanwisher, 2003). Moreover, legal judgments were associated with stronger activation in the left dorsolateral prefrontal cortex, suggesting that this kind of decisions were made with regard to explicit rules and less intuitively than moral decisions (Schleim et al., 2011). In spite of its relevance, moral decision-making remains unexplored in criminal judges or attorneys. Likewise, no study has examined whether the decisions made by these experts are biased by information about the transgressor’s mental state, the use of GL in describing harmful events, or their own physiological states.

To address these issues, we assessed 169 participants, including 45 judges and 60 attorneys specialized in criminal law. On average, judges had been working in criminal law for 19 years (SD = 9.8), whereas attorneys had 13 years (SD = 11.17) of experience as litigators in the field. Outcomes from both groups were compared to those of a control group (*n* = 64) comprised of community members with mixed educational levels, without work experience or a law degree. Of note, whereas previous studies using language manipulations (i.e., gruesome vs. plain language) have focused only on punishment ratings (Treadway et al., 2014), here we investigated the impact of GL on three aspects of moral decision-making: morality (Moll et al., 2005), punishment (Cushman, 2008), and harm severity ratings (Decety and Cowell, 2018; Sousa et al., 2009). These aspects hold great relevance for the present study, since moral judgment is critical for enforcing social norms (Yoder and Decety, 2014) and its neural correlates overlap with those mediating legal decision-making in professional attorneys (Schleim et al., 2011).

**Fig. 1:**
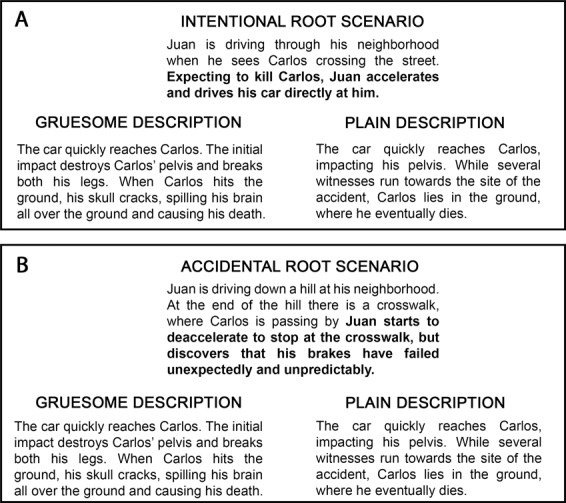
Examples of stimuli for language manipulation. The top panel shows a stem scenario depicting intentional harm. The bottom panel presents a stem scenario depicting accidental harm. At the left side of each panel harm is described with gruesome terms, and at the right side harm is described with plain, just-the-facts language. Note that the consequence in each scenario is the same, namely, death.

Participants completed a modified task (Treadway et al., 2014) consisting of text-based scenarios in which a character inflicts harm on a victim. After reading each story, participants answered three questions by choosing a number from a Likert-like scale using the keyboard (see details in “Methods” section). Participants were asked to (a) rate how morally adequate the transgressor’s action was (morality rating), (b) quantify the amount of punishment the transgressor deserved (punishment rating), and (c) assess the severity of harm that was caused (harm severity rating). The transgressor’s mental state and the situation’s emotional content were manipulated to create four types of scenarios, namely: intentional GL, accidental GL, intentional plain language (PL), and accidental PL scenarios. In half of the scenarios, the main actor deliberately intended the harm that actually befell on the victim (intentional harm). In the remaining half, the actor caused identical damage but without purposeful intent (accidental harm). Additionally, emotional content was manipulated in the scenarios in a between-subjects design. Half of participants were assigned to the GL condition, and the other half to the PL condition. Participants in the GL condition read highly gruesome descriptions of harm, which were intended to amplify emotional reactions (Treadway et al., 2014). Instead, participants in the PL condition read the same stories but were presented with plain, just-the-facts language. Therefore, the actual harm experienced by the victim was equivalent in both conditions (see Fig. 1 for an example).

Also, considering that executive functions (EFs) can modulate moral cognition (Baez et al., 2018; Buon et al., 2016), we examined this domain in a sub-sample of participants (*n* = 86). For instance, moral reasoning maturity is associated with the integrity of EFs (cognitive flexibility, feedback utilization, abstraction capacity, and verbal fluency) (Vera-Estay et al., 2014). In particular, inhibitory control resources enabling regulation and control of other cognitive processes that might be critical for judging accidental harms (Buon et al., 2016). In addition, individual differences in working memory, which reflect cognitive-control variation, predicted moral judgments. Specifically, people with greater working memory abilities perform more rational evaluations of consequences in personal moral dilemmas (Moore et al., 2008). Here, executive functioning was assessed through the INECO frontal screening (IFS) battery (Torralva et al., 2009), a brief and well-validated test in clinical (Baez, Ibanez et al., 2014; Bruno et al., 2015; Torralva et al., 2009) and healthy (Gonzalez-Gadea et al., 2014; Santamaria-Garcia et al., 2019; Sierra Sanjurjo et al., 2019) populations. The IFS assesses various EFs, namely: motor programming, conflicting instructions, inhibitory control, working memory, and abstraction capacity (see details in Supplementary Materials and methods, SI). In addition, given that affective engagement triggered by GL may be indexed by autonomic arousal, we obtained electrocardiogram (ECG) recordings from the same subset of participants in order to examine their heart-rate variability (Castaldo, 2015; Kop et al., 2011; Mccraty et al., 1995; Shaffer and Ginsberg, 2017) during the task.

Given the expertise of judges and attorneys in deciding over transgressions, we expected their moral decisions to be more appropriately adjusted to the perpetrator’s intentions and to rely less on emotional reactions and peripheral physiological signals. In line with these predictions, our results showed that the transgressor’s mental state was a key determinant in moral decision-making (Guglielmo, 2015; Yoder and Decety, 2014). Specifically, we found that, similar to controls, judges and attorneys overestimated the damage caused by intentional harms compared to accidental harms. However, judges and attorneys were less biased towards punishment and harm severity ratings in the face of accidental harm. Also, unlike controls, language manipulations and physiological arousal had no significant effects on judges or attorney’s decisions. Compatibly, morality ratings in response to GL manipulations were predicted by physiological signals only in controls. This suggests that legal decision makers may rely less than controls on physiological signals to evaluate transgressions, although they remain biased by the “harm-magnification effect” (Ames and Fiske, 2013, 2015; Baez, Herrera et al., 2017), which shows that people overestimate the damage caused by intentional harm compared with accidental harm, even when both are identical. Together, these results suggest that specific expertise developed in legal settings can partially abolish strong biases linked to the assessment of others’ mental states, the affective states induced by GL, and the physiological state of one’s own body.

## Results

### Morality ratings

**Fig. 2:**
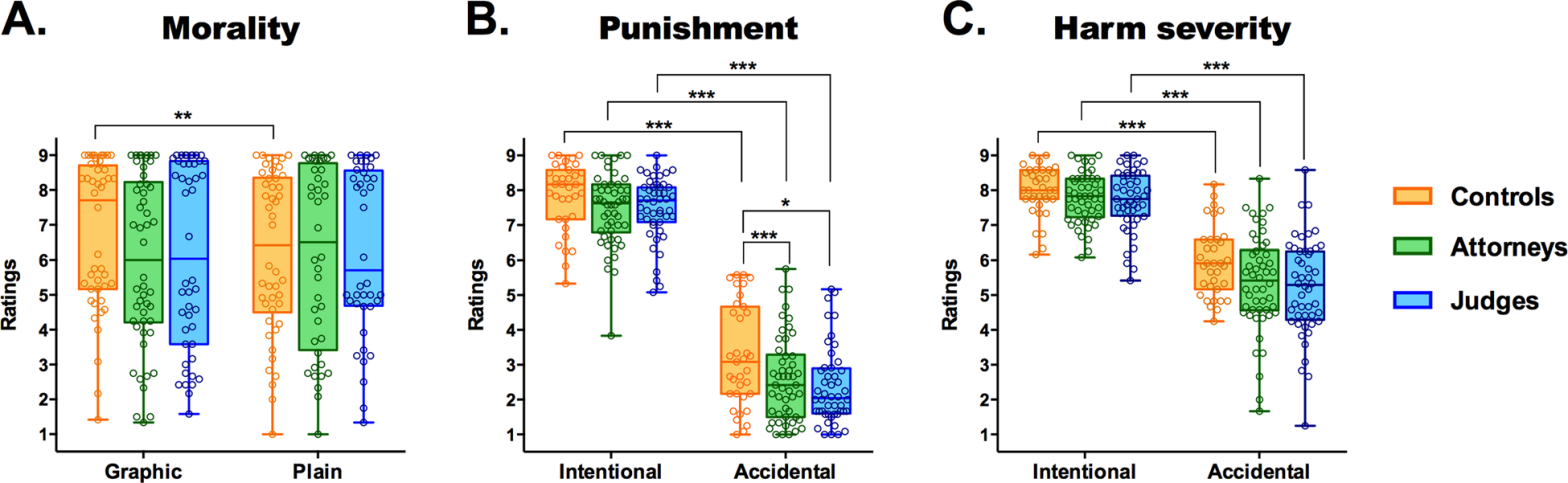
Effects of gruesome language (GL) and intentionality on morality, punishment and damage ratings. **a** We observed a group-by-language interaction, such that only participants of the control group had significantly higher morality ratings when reading gruesome descriptions of harm, relative to the PL condition. **b** We also found a group-by-intentionality interaction, revealing that punishment ratings were significantly lower for the judges and attorneys groups in comparison to controls during accidental scenarios. There were no differences between groups when participants read intentional scenarios. **c** We found a group-by-intentionality interaction, revealing that harm severity ratings were significantly lower for judges and attorneys than controls in accidental scenarios. Participants in all groups assessed harms as significantly greater in magnitude when they were committed intentionally, in comparison to situations when harm was accidentally caused. This reveals a biasing effect of intentionality on damage assessments, because the accidental and intentional conditions contained an equivalent range of harms. Significance coding: *p < 0.01; **p < 0.001; ***p < 0.0001.

In each scenario, participants were asked to judge how morally wrong the protagonist’s transgression was, using a scale from 1 (entirely good) to 9 (entirely wrong). Across groups and PL–GL conditions, participants considered intentional harms as morally worse than accidental ones (*F*_1,163_ = 606.82, *p* < 0.0001, *η*^2^ = 0.78). Also, across groups and accidental and intentional scenarios, participants exposed to GL, compared to those faced with PL, rated harmful actions as morally worse (*F*_1,163_ = 4.77, *p* = 0.03, *η*^2^ = 0.02). Importantly, an interaction was also found between language and group (*F*_2,163_ = 7.16, *p* = 0.002, *η*^2^ = 0.07). Post-hoc comparisons showed that judges and attorneys were immune to the influence of GL, presenting similar morality ratings in both language conditions (judges: *p* = 0.77; attorneys: *p* = 0.50). On the contrary, controls exposed to GL, compared to those faced with PL, rated harmful actions as morally worse (*p* = 0.0002) (Fig. 2a).

### Punishment ratings

Participants also had to decide on the amount of punishment deserved by the transgressor, on a scale from 1 (no punishment) to 9 (severe punishment). Across all groups and language conditions, subjects assigned more punishment to intentional than accidental actions (*F*_1,163_ = 1107.60, *p* < 0.0001, *η*^2^ = 0.87). However, groups behaved differently in their punishment assignment decisions (*F*_2,163_ = 37.85, *p* < 0.0001, *η*^2^ = 0.31). Judges and attorneys punished harmful actions to a similar degree (*p* = 0.98). However, controls punished transgressions more than judges (*p* = 0.00002) and attorneys (*p* = 0.00002). We also found an interaction between intentionality and group (*F*_2,163_ = 17.94, *p* < 0.0001, *η*^2^ = 0.18). The judges (*p* = 0.0002) and attorneys (*p* = 0.00002) assigned significantly less punishment to accidental harmful actions than did controls (Fig. 2b). Moreover, judges and attorneys did not differ in their punishment ratings for the accidental condition (*p* = 0.91). On the other hand, neither judges (*p* = 0.96) nor attorneys (*p* = 0.48) differed from controls in their punishment ratings for intentional harmful actions.

### Harm severity ratings

Finally, participants had to assess how harmful the protagonist’s action was using a scale from 1 (not harmful) to 9 (very harmful). In order to make accidental and intentional conditions comparable, the range of harms was equivalent between them (see “Methods” section). However, across groups and language conditions, participants assigned higher harm severity ratings to intentional than accidental harms (*F*_1,163_ = 170.37, *p* < 0.0001, *η*^2^ = 0.51). Moreover, groups differed in their harm severity ratings (*F*_2,161_ = 10.59, *p* = 0.0004, *η*^2^ = 0.11). Compared to controls (*p* = 0.00003) and attorneys (*p* = 0.040), judges estimated that the transgressor’s actions were less harmful. Attorneys’ damage ratings did not differ from those of controls (*p* = 0.10) (Fig. 2c). We also found an interaction between intentionality and group (*F*_2,163_ = 23.42, *p* < 0.0001, *η*^2^ = 0.2). The judges (*p* = 0.0002) and attorneys (*p* = 0.00002) assigned significantly lower severity harm ratings to accidental harmful actions than did controls (Fig. 2c). Moreover, judges and attorneys did not differ in their harm severity ratings for the accidental condition (*p* = 0.18). Neither judges (*p* = 0.98) nor attorneys (*p* = 0.29) differed from controls in their harm severity ratings for intentional harmful actions. Also, intra-group comparisons showed that the three groups assigned higher harm severity ratings to intentional compared to accidental harms (judges: *p* = 0.00002; attorneys: *p* = 0.00002; controls: *p* = 0.005).

### The role of executive functioning and physiological arousal on moral decisions

We also explored the role of two potential modulators of participants’ decisions via regression models including measures of EFs and heart rate variability (HRV) in a subsample of participants (*n* = 86) comprising 30 attorneys, 27 controls, and 29 judges. Groups in this subsample did not differ in terms of years of education or sex, but they differed significantly in age (see “Methods” section and Table S1). Therefore, to test the potential association between this variable and the measures in which we found group differences, age was included as an additional predictor in all regression models. We estimated the low frequency (LF) band power from participants’ ECGs, given the relevance of this measure as a proxy of emotional arousal (Castaldo, 2015; Kop et al., 2011; Mccraty et al., 1995). We calculated the percentage change of power in this band from baseline to task (Sloan et al., 1995) (see “Methods”; and SI: “Materials and methods”).

#### Morality ratings

The first linear model included group, language, EFs, LF power, and age as predictors, and morality ratings (mean of morality ratings for intentional and accidental harms) as a dependent variable. We used morality ratings averaged over intentionality levels because we previously found that this factor did not interact with group or language. The overall model was statistically significant (*F*_7,78_ = 2.79, *p* = 0.0013, *R*^2^ = 0.22). Age (*t* = −0.034, *p* = 0.20, *β* = −0.02) was not a significant predictor. As expected, the model had a significant group-by-language interaction. This interaction revealed that controls evaluated actions as morally worse compared to judges and attorneys, but only when participants were exposed to GL (GL × attorneys: *t* = −2.85, *p* = 0.0056, *β* = −0.616; GL × judges: *t* = −2.16, *p* = 0.034, *β* = −0.455; PL × attorneys: *t* = −0.44, *p* = 0.66, *β* = −0.133; PL × judges: *t* = −1.34, *p* = 0.18, *β* = −0.291). Such differences between groups in the GL condition were confirmed by follow-up *t*-tests of average morality ratings (attorneys-controls: *t*_25.4_ = −4.18, *p* = 0.0045; Controls-judges: *t*_19.9_ = 2.92, *p* = 0.0084; attorneys-judges: *t*_29.4_ = −0.98, *p* = 0.34; *p*-value adjustment method: Holm–Bonferroni).

**Fig. 3:**
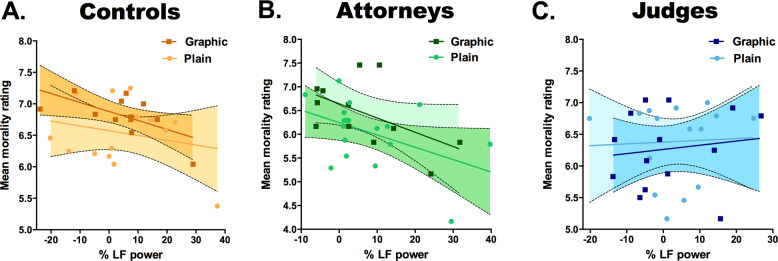
Association between mean morality ratings and emotional activation as indexed by the percentage change of power in the low frequency (LF) band. There was a significant correlation between LF power and mean morality ratings only for control participants that read gruesome descriptions of harm, but not for those that read plain descriptions. **a** This association was not significant in attorneys **b** or judges **c** who read either plain or gruesome descriptions. Depicted in the scatter plots are the regression lines and 95% confidence intervals.

Importantly, LF power predicted average morality ratings in the model (*t* = −2.70, *p* = 0.0086, *β* = −0.06), suggesting that participants may rely on physiological signals to make moral judgments. In order to determine whether this association was present across all groups and language conditions, for each group we ran separate linear simple regressions with average morality as dependent variable and LF power as predictor (Fig. 3). In the GL condition, LF power was not significantly associated to morality ratings made by judges (*t* = 0.47, *p* = 0.64, *β* = 0.13) or attorneys (*t* = −2.06, *p* = 0.06, *β* = −0.45). Contrarily, HRV significantly predicted morality ratings in the GL condition for the control group (*t* = −2.48, *p* = 0.03, *β* = −0.61). HRV was not associated to morality ratings for any group assigned to the PL condition (judges: *t* = 0.17, *p* = 0.86, *β* = −0.04; attorneys *t* = −1.96, *p* = 0.09, *β* = −0.53; controls: *t* = −0.85, *p* = 0.41, *β* = −0.23).

#### Punishment ratings

We fitted an additional linear model including group, EFs, LF power, and age as predictors, and punishment ratings to accidental harms as a dependent variable (the significant variable in previous rating outcomes). Results showed that age (*t* = 0.76, *p* = 0.45, *β* = 0.113), EFs (*t* = −0.47, *p* = 0.64, *β* = 0.01), and LF power (*t* = −1.89, *p* = 0.062, *β* = 0.04) were not significant predictors of punishment ratings, although the overall model was statistically significant (*F*_6,79_ = 3.41, *p* = 0.0048, *R*^2^ = 0.15). Regarding group differences, only the slope of the judges’ group was significant (attorneys: *t* = −1.51, *p* = 0.14, *β* = −0.37; judges: *t* = −4.04, *p* = 0.00012, *β* = −1.03; controls were the reference group). Therefore, punishment assignment results do not appear to be explained by age, HRV or EFs.

#### Harm severity ratings

Lastly, we fitted a linear model to assess the contribution of EFs, HRV, and age on harm severity ratings. This model included group, age, EFs scores, and LF power as predictors, with harm severity ratings to accidental harms (the relevant outcome of previous rating results) as the dependent variable. Even though the overall model was significant (*F*_6,79_ = 2.89, *p* = 0.026, *R*^2^ = 0.12), only EFs scores significantly predicted mean harm severity ratings (*t* = 2.37, *p* = 0.01, *β* = 0.25). No other significant associations were observed.

## Discussion

To our knowledge, the present study represents the first experimental comparison of moral decision-making in criminal judges, attorneys, and controls, focusing on three sources of bias: (a) information about the transgressor’s mental state, (b) language manipulations aimed at provoking emotional reactions, and (c) ongoing physiological states. We found that information on the transgressor’s mental state influenced morality, punishment, and harm severity ratings across all groups. However, judges and attorneys ascribed significantly less punishment and harm severity ratings to accidental harms than did controls. Moreover, the decisions of judges or attorneys were not biased by GL or ongoing physiological signals in the face of harmful actions. Together, these results indicate that academic background and professional expertise can shape the minds of legal decision makers, illuminating the potential role that legal expertise may have in overriding cognitive, emotional, and physiological biases lurking behind their daily work.

Information on the transgressor’s mental state influenced moral decision-making across all groups. Our results confirmed that, compared to accidental harms, intentional ones were evaluated as morally worse (Cushman, 2008; Young and Saxe, 2008), received harsher punishments (Buckholtz et al., 2015; Cushman, 2008), and were considered more damaging (Ames and Fiske, 2013, 2015). However, judges and attorneys ascribed significantly less punishment to accidental harms than did controls, there being no between-group differences for intentional harms. Previous evidence from healthy (Decety et al., 2012) and clinical (Baez et al., 2014, 2016) populations shows that intentionality comprehension is higher for intentional than accidental harm, suggesting that the latter is less clear or explicit and involves greater cognitive demands (Baez et al., 2016). Also, it has been suggested that a robust representation of the other’s mental state is required to exculpate an accidental harm (Young et al., 2007; Young and Saxe, 2009b). This robust representation allows overriding a preponderant response to the salient information about actual harm. Thus, the present results suggest that legal experts may be more skilled at detecting the intentionality of the actor, representing his/her mental state, and overriding the prevalent response to the outcome. In law, blameworthiness is judged, among other factors, by reference to the mental state that accompanied a wrong action (Buckholtz and Faigman, 2014). Punishment for a harmful action hinges on a determination of moral blameworthiness (in criminal contexts) or liability (in the law of torts) (Buckholtz and Faigman, 2014). Such determinations require inferences about the beliefs, intentions, and motivations of the individual being considered for sanction (Buckholtz and Marois, 2012). Thus, our findings suggest that the expertise of judges and attorneys could hone intentionality detection abilities, leading to more objective punishment ratings. However, as we did not include specific measures of intentionality detection abilities, future studies on legal decision-makers should test this interpretation.

Although, compared to controls, judges and attorneys assigned lower harm severity ratings to accidental harms, across groups and language conditions, participants assigned higher ratings to intentional compared to accidental harms. Since harm severity was identical in both intentionality conditions, our results imply that judges and attorneys are also biased by the widely described “harm-magnification effect” (Ames and Fiske, 2013, 2015): people overestimate an identical damage when intentionally inflicted. Studies on defensive attributions (Shaver and Drown, 1986), retributive justice (Darley and Pittman, 2003), and moral psychology (Knobe et al., 2012) converge to show that when people detect harm, they are urged to blame someone. However, people are notoriously more sensitive to harmful intentions. Indeed, the urge to find a culprit is higher in the face of intentional than accidental harms (Ames and Fiske, 2015; Young and Saxe, 2009a). Such motivation to blame causes people to overestimate actual damage (Ames and Fiske, 2013). This view aligns with traditional philosophical accounts (Nagel, 1979; Williams, 1982) suggesting that “moral luck” reflects the direct influence of the outcome on moral judgments. The perceived severity of harmful outcomes can influence moral judgments independently of inferences that people make about a harmful actor’s beliefs or desires (Martin and Cushman, 2016). Thus, bad outcomes would lead directly to more blame, independent of other facts about the agent and the action (Zipursky, 2008). Given this motivation to blame harm-doers, people emphasize evidence that make their case more compelling (Alicke and Davis, 1990), and one tactic to do this would be to imply that harm-doers caused more harm than they actually did (Ames and Fiske, 2013). Thus, harmful acts that lead to especially large amounts of blame motivation also lead to exaggerated perceptions of harm.

An additional explanation to this effect is the fact that intentional harm (as opposed to accidental harm) involves an additional “symbolic” damage to the victim (Darley and Huff, 1990), beyond the physical injury or damage to property. In particular, the sensitivity of judges and attorneys to this effect could reflect their training to recognize that these additional consequences, rather than the harmful result by itself, may be derived from an intentional harm. Compared to accidental harms, intentional harms may result in more important subjective losses, such as pain, suffering or emotional distress for the victim. The specific knowledge about law might explain the overestimation of intentional harm by judges and attorneys. Still, this finding has important implications, since the harm-magnification effect may inflate legal sentences. In sum, our findings suggest that legal expertise might improve intentionality detection abilities without abolishing the harm-magnification effect. This speaks to a partial discrepancy between the way humans actually make decisions and the underlying assumptions of the legal system.

We also manipulated the emotional responses to harm by describing scenarios via GL and PL (Treadway et al., 2014). Results showed that GL did not bias the judges’ and attorneys’ decisions, suggesting that expertise and academic background in criminal law render these individuals more immune to the effects of language bias on moral decision-making. On the contrary, GL biased morality ratings in controls, supporting similar reports in moral decision-making (i.e., punishment ratings in controls) (Treadway et al., 2014) and in mock-jurors’ decisions (Bright and Goodman-Delahunty, 2006, 2011; Whalen and Blanchard, 1982). Thus, our findings suggest that legal system experts are not biased by language-triggered emotional reactions, even when these effects impact ordinary citizens (who, in some countries, can actually act as jurors in legal settings).

No effects of language manipulations were found on punishment or harm severity ratings. Note that, as opposed to these, morality ratings seem to be more automatic (Haidt, 2001) and precede punishment and harm severity decisions (Buckholtz et al., 2008). Punishment decisions seem to be less automatic and require the integration of mental state information and harm severity assessment (Buckholtz et al., 2015; Carlsmith et al., 2002). Unlike the latter, morality decisions involve an instant feeling of approval or disapproval when witnessing a morally-laden situation (Haidt, 2001) as well as evaluative judgments based on socially shaped ideas of right and wrong (Moll et al., 2005). Note that, while previous research using language manipulations (i.e., gruesome vs. plain language) has only focused on punishment ratings (Treadway et al., 2014), ours is the first investigation on the impact of GL on morality, punishment, and harm severity ratings. Thus, our results suggest that when different dimensions of moral decision-making are assessed, automatic emotional-triggering biases (such as those linked to GL) could affect morality ratings more than punishment and harm severity ratings.

We performed complementary analyses to explore the effect of crime type on the observed group differences. We found that across morality, punishment, and harm severity dimensions, participants assigned higher ratings to death compared to property damage scenarios. For morality and harm severity, participants also provided higher ratings to death compared to physical harm scenarios. These results are consistent with those of previous studies showing that the magnitude of harm (i.e., actions resulting in death versus loss of property) predicts higher morality (Gold et al., 2013) and punishment (Treadway et al., 2014) ratings. Importantly, these differences are present across the three groups and do not explain the observed effects of language in controls’ morality ratings or the group effects on punishment and harm severity ratings for accidental harms.

We also explored the effects of years of experience in criminal law on moral decision-making of judges and attorneys. The former variable was not significantly associated with morality, punishment or harm severity ratings in either judges or attorneys. It is worth noting that years of professional practice was the only measure of experience included in this work. Future studies should further investigate whether specific components of experience, such as levels of exposure or desensitization, contribute to moral decision patterns observed in these populations. Our findings suggest that, rather than years of experience, criminal law expertise (specific knowledge, background, and technical skills), and the professional role per se (judges and attorneys) seem to have a more relevant role in overriding cognitive and emotional biases, which can influence moral decision-making. In line with our results, previous evidence has shown that expertise and experience may play different roles on judicial decision-making. For instance, prior expertise enhances the influence of ideology on judicial decision-making, but accumulated experience does not (Miller and Curry, 2009). Legal experts with domain-specific expertise in criminal law show less sensitivity to confirmatory bias than legal professionals without this expertise (with specializations in other fields than criminal law) (Schmittat and Englich, 2016). Thus, our findings and previous evidence suggest that specific knowledge, background, and technical skills in criminal law have a relevant role in overriding cognitive and emotional biases which can influence decision- making.

It is worth noting that judges were the only group whose physiological signals showed no association with decision patterns. This relation was marginally significant in attorneys and fully significant in controls (with HRV predicting morality ratings in the GL condition). Consistent with the results of language manipulation, moral decision-making in judges and, to a less degree, in attorneys, seems to rely less on peripheral physiological signals usually associated with emotional reactions (Kop et al., 2011; Mccraty et al., 1995; Shaffer and Ginsberg, 2017). This pattern suggests that the particular expertise and training of judges and attorneys is relevant in reducing the effect of physiological arousal on moral decision-making. Both, judges and attorneys are repeatedly exposed to graphic or gruesome material and this could reduce the associated physiological arousal. Besides, both groups have specific academic background on the ideal of non-biased decision-making, and their everyday activities may provide them with training in identifying and avoiding biases associated to physiological signals. As no measures of exposure to gruesome material in professional practice or explicit knowledge on biases associated with decision-making were included in this study, future research should test these interpretations. Note that the association between physiological signals and morality ratings is completely absent only in judges. This difference between judges and attorneys may be explained by the specific role that each one plays in their daily practice. Unlike attorneys, judges preside or decide at trials, decide what evidence will be allowed, and instruct juries on the law they should apply. Thus, it is expected that judges should be impartial and decide according to the law, free from the influence of biases. Conversely, attorneys represent and defend their clients’ position. Therefore, although both judges and attorneys are repeatedly exposed to gruesome material, only the first are expected to impartially evaluate and decide on this evidence. These differences may explain why the reliance of moral decisions on peripheral physiological signals was greater for attorneys than judges. This hypothesis should be directly assessed in future studies.

Besides, our results showing that in controls physiological signals triggered by GL are associated with morality ratings support previous studies showing that gruesome evidence provokes emotional reactions (Bright and Goodman-Delahunty, 2006; Salerno and Peter-Hagene, 2013; Treadway et al., 2014) and boosts the activity of the amygdala, a key brain region involved in emotional processing and harm encoding (Bright and Goodman-Delahunty, 2006; Hesse et al., 2016; Salerno and Peter-Hagene, 2013; Shenhav and Greene, 2014; Treadway et al., 2014). Thus, our study supports empirical (Damasio, 1994; Greene and Haidt, 2002; Haidt, 2008; Moll et al., 2005) and theoretical (Damasio, 1994; Forgas, 1995; Haidt, 2001) claims that bodily and emotional reactions impact on moral decision-making. In addition, our results suggest that these reactions can be attenuated by legal expertise.

Finally, we found that executive functions (EFs) significantly predicted mean harm severity ratings, confirming the role of these domain-general skills in moral decisions (Baez et al., 2018; Buon et al., 2016). Tentatively, this indicates that EFs may support the regulation and control of diverse cognitive processes critical for moral judgment (Buon et al., 2016). Further research using more extensive assessments should identify specific relationship between EFs and different aspects of moral decision-making in expert and non-expert populations.

Our findings may have important implications for context-based modulations of cognitive processes (Baez et al., 2017; Barutta et al., 2011; Cosmelli and Ibáñez, 2008; Ibáñez et al., 2017; Ibañez and Manes, 2012; Melloni et al., 2014) underlying legal behavior. Morality is a fundamental component of human cultures, affording a mechanism for social norm enforcement (Yoder and Decety, 2014). Indeed, morality hinges on prescriptive norms about how people should treat one another, including concepts such as justice, fairness, and rights (Yoder and Decety, 2014). Yet, in addition to ordinary moral norms, law exerts an additional regulatory role in social life (Schleim et al., 2011). Indeed, neuroimaging studies have shown similarities between neural basis of moral and legal decision-making in professional attorneys, suggesting a considerable overlap in cognitive processing between both normative processes (Schleim et al., 2011). These partial overlaps between moral and legal decision-making highlight the potential translational implications of our results. The legal system must regulate sources of bias in defendants, jurors, attorneys, and judges (Greely, 2011). Our results provide unique evidence that judges and attorneys are less impacted by typical biases in third-party morally laden decisions. Thus, results support a “bias-reduced” approach to law (Gewirtz, 1996), at least regarding the effects of language manipulations and associated physiological signals. Our results may have implications in countries that use juries as part of their legal system. Although it has been questioned whether judges should withhold relevant evidence from jurors, fearing that they would use it in an impermissible manner (e.g., Pettys, 2008), our results provide empirical support to formal postulates such as the following one, from the United States Federal Rules of Evidence: “*The court may exclude relevant evidence if its probative value is substantially outweighed by a danger of one or more of the following: unfair prejudice, confusing the issues, misleading the jury, undue delay, wasting time, or needlessly presenting cumulative evidence”*. Endorsing this rule, our results suggest that ordinary citizens (who can be potential jurors) are more biased than judges in the face of language manipulations and associated physiological states.

In addition, we found that judges and attorneys are not impervious to the “harm-magnification effect”: just like controls, these experts overestimated the damage caused by intentional harms (Ames and Fiske, 2013, 2015; Darley and Huff, 1990). This result may have important implications, since the harm-magnification effect may inflate legal sentences (Ames and Fiske, 2013; Darley and Huff, 1990). Indeed, intentional damage to property is judged as more expensive than accidental damage (Ames and Fiske, 2013; Darley and Huff, 1990). This finding is in line with previous reports showing that decisions of legal experts may be affected by several biases (Englich et al., 2006; Guthrie et al., 2001; Rachlinski et al., 2008, 2015; Wistrich et al., 2015). Also, our result aligns with the suggestion (Burns, 2016; Tsaoussi and Zervogianni, 2010) that judicial decisions are not immune to the impact of “bounded rationality”. This term refers to the concept that “human cognitive abilities are not infinite” (Simon, 1955) and, therefore, people take short-cuts in decision-making which may not be considered rational. Thus, the bias towards overestimation of damage caused by intentional harm is an important issue that should be explicitly acknowledged in legal settings or even in law instruction programs. Indeed, it has been shown that although judges may carry racial biases, they are able to suppress them when motivated and explicitly instructed to monitor their own implicit biases (Rachlinski et al., 2008).

In conclusion, this is the first study to examine whether different dimensions (morality, punishment, and harm severity) of judges and attorneys’ decisions are biased by information about the transgressor’s mental state, the use of GL in describing harmful events, or their own physiological states. We found that judges and attorneys’ decisions are not affected by the use of GL or physiological signals, and are accurately sensitive to the information on the transgressor’s mental state. Judges and attorneys seem to be more skilled than controls at identifying accidental harms, which contribute to more fair punishment assignments. However, judges and attorneys are not immune to the harm-magnification effect. Our results offer new details about how expertise can shape the minds of legal decision makers, paving the way for promising new research into the cognitive and physiological factors associated with legal decision-making. Present results could inspire new ecological designs tracking the potential effects of the transgressor’s mental state, language manipulations, and physiological signals in legal decision makers.

## Methods

### Participants

One hundred and sixty-nine participants took part in the study. The judges’ group included 45 subjects who had held the position of judge in the field of criminal law (mean age = 44.17, SD = 8.98). The attorneys’ group included 60 attorneys with experience in litigation in the field of criminal law (mean age = 37.06, SD = 9.98). Three attorneys had received graduate education in criminal law. On average, participants in the judges’ group had 19.09 (SD = 9.81) years of work experience in criminal law, whereas attorneys had 13 years (SD = 11.17). Sixty-four community members with a mixed educational background (mean age = 41.39, SD = 11.84) were recruited for the control group. All of them lacked a law degree, professional qualifications in the field, and work experience related to criminal law. The three groups did not differ statistically in terms of sex (chi-squared = 0.44, *p* = 0.79). However, there were group differences in terms of years of education (*F*_2,166_ = 11.65, *p* = 0.00002) and age (*F*_2,166_ = 6.20, *p* = 0.02). Controls had significantly fewer years of education than judges (*p* = 0.00003) and attorneys (*p* = 0.001), but no difference was found between the latter two groups (*p* = 0.39). Regarding age, attorneys were significantly younger than judges (*p* = 0.001), but controls did not differ from judges (*p* = 0.35) or attorneys (*p* = 0.07). All participants were native Spanish speakers. Participants with visual disabilities, history of substance abuse, and neurological or psychiatric disorders were excluded.

The study included participants from Colombia and Argentina. We obtained measurements of general cognitive state, executive functioning (see Table S1 and Materials and methods SI), and ECG recordings from a subsample of Colombian participants (*n* = 86). This subsample included 30 attorneys, 27 controls, and 29 judges and was assessed individually in an isolated office. The remaining participants (*n* = 83) completed the experiment online (see Materials and methods SI*)*.

The three groups of this subsample did not differ statistically in terms of years of education, sex, global cognitive functioning, and executive functioning (see Table S1). Nevertheless, attorneys were significantly younger than judges and controls. Therefore, we calculated mixed ANCOVA models for all ratings including years of education and age as a covariate.

### Procedure

The study was approved by the institutions’ ethical committees and conducted in accordance with the Declaration of Helsinki. All participants provided informed consent prior to the experimental procedures, as well as relevant information such as socio-demographic data, past job experience, and medical antecedents. After that, participants undertook the experiment individually.

#### Moral decision-making task

Participants completed a modified computerized version of a task tapping moral evaluation, punishment assignment, and harm assessment (Treadway et al., 2014). The instrument comprised 24 core scenarios involving two characters: a protagonist that inflicted harm and a victim that suffered that harm. Here, harm refers to physical damage to people or property. Specifically, the text-based scenarios varied in terms of the degree of harm, and were divided into three categories: property damage, physical harm (assault or maiming), and death. From each stem scenario, we employed four variation scenarios that differed in the intentionality of the transgressor (accidental vs. intentional) and the language used to describe harm (gruesome vs. plain).

The four scenario variations were the following: intentional harm/plain language (intentional-PL), accidental harm/plain language (accidental-PL), intentional harm/gruesome language (intentional-GL), and accidental harm/gruesome language (accidental-GL). Each subject read a given stem scenario only once. Participants assigned to the PL condition read only scenarios with descriptions of harm in PL. On the contrary, participants assigned to the GL condition read only scenarios describing harm through gruesome terms. Critically, the GL and PL conditions were identical except for the language used to describe harm (see Fig. 1 for an example of the language manipulation).

All participants read 12 intentional and 12 accidental scenarios, which were presented in a pseudorandomized order. With the objective of mitigating possible order effects, we counterbalanced the presentation of intentional and accidental scenarios across participants. Therefore, there were four versions of the task in total, two for each language condition and, within each language condition, two versions that reversed the order of accidental and intentional scenarios. Such counterbalancing of intentional and accidental scenarios guaranteed that the degree of harm was equivalent between the accidental and intentional conditions across participants. In summary, this experiment consisted of a 2 × 2 × 3 design, with language and group as the between-subjects factors, and intentionality as the within-subjects factor.

After reading each story, participants answered three questions by choosing a number from a Likert-like scale using the keyboard. In the first question, participants were asked to rate how morally adequate the transgressor’s action was (morality rating, 1 = “entirely wrong”, 9 = “entirely good”). To analyze the data, we inverted this scale to make the comparison between ratings more intuitive. Thus, for reported results, morality ratings ranged from 1 (“entirely good”, 9 = “entirely wrong”). The second required participants to quantify the amount of punishment the transgressor deserved (punishment rating, 1= “no punishment”, 9= “severe punishment”). The final question asked participants to assess the severity of harm that was caused (how harmful was the action? harm severity rating, 1 = “no harm”, 9 = “very harmful”).

Effects of intentionality, language, and crime type were tested in an initial pilot study conducted to validate our materials (see Supplementary methods, SII). Results of this pilot study showed that morality, punishment, and harm severity ratings were higher for intentional harms than accidental ones. GL showed a significant effect only on morality ratings. Regarding the types of crime, across morality, punishment, and harm severity, participants assigned higher ratings to death compared to property damage scenarios. For morality and harm severity, participants also provided higher ratings to death compared to physical harm scenarios. For harm severity, ratings were also higher to physical harm than property damage.

#### Behavioral data analysis

Behavioral data (morality, punishment, and damage ratings) were analyzed using R version 3.5.2. All statistical tests used were two-sided, unless explicitly stated. The significance level was set at 0.05 for all tests. To assess the potential interactions between group, language, and intentionality, we employed mixed ANOVAs. The generalized eta-squared was used as a measure of effect size. Normality of studentized residuals of these models was evaluated using quantile–quantile plots and the Shapiro–Wilk test. Since the assumptions of normality and homogeneity of variances were not met, we transformed morality and harmfulness ratings by applying the Box–Cox power transformations (Box and Cox, 1964; Sakia, 1992). A maximum-likelihood procedure allowed us to estimate the lambda coefficients of those transformations. Such transformations increased the fit of the studentized residuals to a normal distribution and also proved to stabilize variance.

Furthermore, given that groups differed in terms of age and years of education, and that these two variables may have an effect on moral decision-making (Al-Nasari, 2002; Krettenauer et al., 2014; Maxfield et al., 2007; Rosen et al., 2016), we calculated mixed ANCOVA models for all ratings, taking group, language, and intentionality as factors, and age and years of education as covariates. We reported *p*-values and statistics from the post-hoc test of the mixed ANCOVA models. Normality and homoscedasticity criteria were not fully met even after data transformation. Therefore, we verified all ANOVA results using the Welch–James statistic for robust testing under heterocedasticity and non-normality, with 0.2 mean trimming, Winsorized variances, and bootstrapping for calculating the empirical critical value (Keselman et al., 2003; Villacorta, 2017; Wilcox, 2011). Results were almost identical to those of the mixed ANOVA models (see Results SI). To further decompose significant interactions and evaluate significant main effects, we employed Tukey-adjusted pairwise comparisons of least-square means as a post-hoc test for the mixed ANOVAs. In addition, follow-up tests for significant interactions were verified with planned comparisons using a non-parametric test (Wilcoxon), with Holm–Bonferroni adjustment for multiple comparisons. Results of those non-parametric follow-up tests were virtually the same to the post-hoc contrasts of the mixed ANOVA models.

A power analysis showed that with an effect size of 0.25, *α* = 0.05, and a power of 80%, a sample size of 158 participants was required. This assumption was met, since behavioral data analyses were performed on 169 participants, yielding a power of 0.83.

In addition, to explore the association between years of work experience in criminal law of judges and attorneys and moral decision-making, we calculated three linear regression models that included this variable as predictor. Group (judges and attorneys), language, and age were also included as predictors. The models encompassed average morality (morality ratings averaged over intentionality conditions), accidental punishment (punishment ratings in response to accidental harms), and severity ratings for accidental harms as dependent variables.

#### Physiological data analysis

From participants’ ECG recordings, we extracted the LF (0.04–0.15 Hz) power component of HRV (see SI: Materials and methods for details). We calculated LF power during the baseline period (5 min). Also, we estimated LF power over several contiguous 5-min recording windows during the task, and then computed the average power in this band across the windows (García-Martínez et al., 2017). Importantly, groups did not differ in the length of the task recordings (control, mean duration = 1325.4 s, SD = 306.8; attorneys, mean duration = 1401.4 s, SD = 309.2; judges, mean duration = 1411.3 s, SD = 313.7; *F*_2, 83_ = 0.53, *p* = 0.53). Given that the distribution of LF power was highly skewed, we log-transformed this variable to diminish the impact of outlying observations (Electrophysiology, 1996).

Power in the LF band is primarily generated by the vagal control of heart function (Billman, 2013; Goldstein et al., 2011; Reyes Del Paso et al., 2013), and provides information about blood pressure regulatory mechanisms (Goldstein et al., 2011; Reyes Del Paso et al., 2013). Moreover, LF power proves sensitive to emotional activation (Castaldo, 2015; Kop et al., 2011; Mccraty et al., 1995). In particular, psychological stress is associated to a LF power reduction when the task involves movement of the hands to control a keyboard (Hjortskov et al., 2004; Taelman et al., 2011; Yu and Zhang, 2012). In consequence, we expected that LF power would diminish with increments in arousal, which presented an opportunity to test our primary hypothesis concerning GL (Bright and Goodman-Delahunty, 2006; Treadway et al., 2014).

To understand the association between LF power, EFs, and age on group differences during the task, we calculated linear regression models that included those variables as predictors. We computed the percentage change of LF power, from baseline to task, to standardize this measure for each participant. Age was included in those models to control for significant age differences among groups (see Supplementary Table S1). The models included average morality (morality ratings averaged over intentionality conditions), accidental punishment (punishment ratings in response to accidental harms), and mean damage (damage ratings averaged over intentionality conditions) as dependent variables. In the three multivariate linear regression analyses controls were used as the reference group. Also, GL was the reference condition in the average morality model. We transformed dependent variables by applying the Box–Cox power transformations, to increase the fit of the models’ residuals to a normal distribution. A maximum-likelihood procedure allowed us to estimate the lambda coefficients of each transformation.

A second power analysis showed that with an effect size of 0.25, *α* = 0.05, and a power of 80%, a sample size of 79 participants was required for these multiple regression analyses. This assumption was met, there were performed on a subsample of 86 participants, yielding a power of 0.95.

### Supplementary information

Supporting information

## Data Availability

The data that support the findings of this study are available from the corresponding author upon reasonable request.
