# Supplementary material for: The impact of legal expertise on moral decision-making biases
Source: Humanit Soc Sci Commun. 2020 Sep 23;7(1):103. doi: 10.1057/s41599-020-00595-8 (PMC11230913; doi:10.1057/s41599-020-00595-8)
Supplement: Supplementary file 1 — Supporting information [file 41599_2020_595_MOESM1_ESM.docx]

**The impact of legal expertise on moral decision-making biases**

Sandra Baez, Michel Patiño-Sáenz, Jorge Martínez-Cotrina, Diego Mauricio Aponte, Juan Carlos Caicedo, Hernando Santamaría-García Daniel Pastor, María Luz González-Gadea, Martín Haissiner, Adolfo M. García, Agustín Ibáñez

**SUPPORTING INFORMATION**

**SI. MATERIALS AND METHODS**

**Experimental setting.** All Argentinean participants (*n* = 65) and some Colombian participants (*n* = 18) completed the experiment online. The invitation to participate was sent via e-mail, along with the link containing the informed consent, the experimental task, and a questionnaire. We employed the Qualtrics platform for this purpose.

**Psychological tests.** Prior to the experiment, we assessed the participants’ global cognitive state and executive functioning in a subsample of Colombian participants (*n* = 86). Global cognitive state was measured using the Montreal Cognitive Assessment (MoCA) test (Julayanont et al., 2014). Executive functioning was assessed through the INECO Frontal Screening (IFS) battery (Torralva et al., 2009), which is a brief and well-validated (Baez et al., 2014; Bruno et al., 2015; Gleichgerrcht et al., 2011; Moreira et al., 2014) test comprising eight subtests tapping into various executive function domains, namely: (i) motor programming (Luria series, “fist, edge, palm”); (ii) conflicting instructions (subjects are asked to hit the table once when the administrator hits it twice, or vice versa); (iii) motor inhibitory control; (iv) numerical working memory (backward digit span); (v) verbal working memory (months backwards); (vi) spatial working memory (modified Corsi tapping test); (vii) abstraction capacity (inferring the meaning of proverbs); and (viii) verbal inhibitory control (modified Hayling test). The maximum possible score on the IFS is 30 points.

**ECG acquisition.** Heart rate was monitored through an electrocardiogram (ECG) recording. We attached three disposable pre-gelled electrodes (${3M}^{TM}$ foam monitoring electrodes (2228), 3M, United States) to participants in an Einthoven’s triangle configuration. We acquired the electrophysiological signal using a 16/35 Powerlab unit device connected to a dual BioAmplifier (ADinstruments, Colorado Springs, CO), at a 2000Hz-sampling rate (Task Force of the European Society of Cardiology and the North American Society of Pacing and Electrophysiology, 1996). The raw signal was processed online in this device using the following analog filters: A) a 0.02-500 Hz band-pass filter, B) a notch filter (60 Hz) and C) a Mains adaptive filter (7–10).

**Heart rate variability (HRV).** We used the software LabChart pro version 7.3.7 (ADinstruments, Colorado Springs, CO) to further process and analyze ECG recordings. The aim was to calculate a particular measure of the beat-to-beat variation of heart rate (heart rate variability; HRV) during the baseline period, and also during the experimental task. In the present study, we computed the low frequency (LF; 0.04 to 0.15 Hz) power component of HRV.

Prior to calculating the LF power, we first applied various digital FIR filters to the ECG signal to reduce the impact of physiological and technical perturbations. The filters were designed to approximate: A) a fourth order low-pass Butterworth filter, B) a second order high-pass Butterworth filter and C) a notch filter (60 Hz) (Hejjel, 2004; Ruha et al., 1997). Subsequently, an algorithm automatically detected the QRS complexes in the recordings, from which R-R intervals were computed (ms). Such automatic identification of R spikes was corroborated by careful visual inspection and, if an error was detected, the misplaced marker and the associated R-R intervals were eliminated from the analysis. Segments containing ectopic beats were also removed from the analysis. The resulting R-R time series were used to estimate the power spectral density by applying the method of the Lomb-Scargle periodogram (Clifford and Tarassenko, 2005). We made sure that every interval over which LF power was calculated had the minimum number of beats to fulfill the Nyquist constrain. In addition, we did not admit into the analysis recoding segments that had lost more than 20 percent of the R-R intervals due to artifacts or physiological perturbations (Clifford and Tarassenko, 2005).

**SII. SUPPLEMENTARY METHODS**

**Pilot study.** One hundred and fifteen healthy volunteers (63 women, 52 men) participated in the pilot study. Participants completed an online version of the moral decision-making task described in the Procedure section. Participants had a mean of 35.62 (*SD* = 11.7) years of age and 18.50 (*SD* = 5.18) years of education. To assess the effects of intentionality, language, and crime type on each morality rating, we employed mixed 2 x 2 x 3 ANOVAs.

**Results**

**Morality ratings.** Across crime types and language conditions, participants considered intentional harms as morally worse than accidental ones (*F_1,113_* = 8.81, *p* < 0.003, η^2^ = 0.07). Also, across crime types and accidental and intentional scenarios, participants exposed to GL, compared to those faced with PL, rated harmful actions as morally worse (*F_1,113_* = 58.59, *p* > 0.00001, η^2^ = 0.34). Across language conditions and accidental and intentional scenarios (*F_2,224_* = 7.14, *p* = 0.0009, η^2^ = 0.06), participants rated death scenarios as morally worse than physical harm (*p* = 0.009) and property damage (*p* = 0.0006). No difference was observed between ratings for physical harm and property damage (*p* = 0.72).

There were no significant interactions between intentionality and language (*F_1,113_* = 2.00, *p* = 0.15, η^2^ = 0.01), intentionality and crime type (*F_2,224_* = 0.80, *p* = 0.45, η^2^ = 0.007) or between crime type and language (*F_2,224_* = 2.42, *p* = 0.09, η^2^ = 0.02).

**Punishment ratings.** Across crime types and language conditions, participants punished intentional harms more than accidental ones (*F_1,113_* = 19.86, *p* = 0.00002, η^2^ = 0.15). Moreover, across language conditions and accidental and intentional scenarios (*F_2,224_* = 4.39, *p* = 0.01, η^2^ = 0.03), participants assigned more punishment to death compared to property damage scenarios (*p* = 0.02). No difference was observed between ratings for death and physical harm (*p* = 0.07) or physical harm and property damage (*p* = 0.88). No significant main effect of language was observed (*F_1,113_* = 0.72, *p* = 0.39, η^2^ = 0.006).

There were no significant interactions between intentionality and language (*F_1,113_* = 1.59, *p* = 0.20, η^2^ = 0.01), intentionality and crime type (*F_2,224_* = 0.70, *p* = 0.40, η^2^ = 0.007) or between crime type and language (*F_2,224_* = 1.63, *p* = 0.19, η^2^ = 0.01).

**Harm severity ratings.** Across crime types and language conditions, participants assigned higher harm severity ratings to intentional than accidental ones (*F_1,113_* = 5.79, *p* = 0.01, η^2^ = 0.04). Furthermore, across language conditions and accidental and intentional scenarios (*F_2,224_* = 22.31, *p* > 0.00001, η^2^ = 0.16), participants assigned higher ratings to death compared to physical harm (*p* = 0.0003) and property damage scenarios (*p* = 0.00002). Participants also assigned higher ratings to physical harm compared to property damage (*p* = 0.02). No significant main effect of language was observed (*F_1,113_* = 2.46, *p* = 0.11, η^2^ = 0.02).

There were no significant interactions between intentionality and language (*F_1,113_* = 1.35, *p* = 0.24, η^2^ = 0.01), intentionality and crime type (*F_2,224_* = 2.35, *p* = 0.09, η^2^ = 0.02), or between crime type and language (*F_2,224_* = 0.98, *p* = 0.37, η^2^ = 0.008).

**SI. SUPPLEMENTARY RESULTS**

Since distributional assumptions were not fully met even after data transformation, we verified all mixed ANOVAs main effects and interactions with robust testing under non-normality and heterocedasticity. The Welch-James statistic with 0.2 mean trimming, Winsorized variances and bootstrapping for calculating the empirical critical value yielded different results only for the transformed damage ratings. Specifically, we found that gruesome language significantly increased participants damage estimations (*WJ_2,73.40_* = 5.15, *p* = 0.022). Nevertheless, since we did not find such effect using the mixed ANOVA models, we chose not to report that result given the lack of convergence between tests.

**SII. SUPPLEMENTARY RESULTS**

**Morality ratings**

To explore whether crime type had an effect on the group differences observed in morality ratings (averaged over intentionality conditions), we conducted a 3 × 3 x 2 ANOVA, with crime type (3) as a within-subjects factor, and group (3) and language (2) as between-subjects factors. Results showed that across groups and language conditions (*F_2,328_* = 27.84, *p* > 0.00001, η^2^ = 0.14), participants rated death scenarios as morally worse than those involving physical harm (*p* = 0.00002) and property damage (*p* = 0.0002). No difference was observed between ratings for physical harm and property damage (*p* = 0.16). Also, across groups and crime types, participants exposed to GL, compared to those faced with PL, rated harmful actions as morally worse (*F_1,163_* = 5.01, *p* = 0.03, η^2^ = 0.02). Importantly, there was an interaction between language and group (*F_2,163_* = 5.39, *p* = 0.005, η^2^ = 0.06). Post-hoc comparisons showed that judges and attorneys were immune to the influence of GL, presenting similar morality ratings in both language conditions (Judges: *p* = 0.99; attorneys: *p* = 0.99). On the contrary, controls exposed to GL, compared to those faced with PL, rated harmful actions as morally worse (*p* = 0.0004).

There were no significant interactions between crime type and group (*F_4,328_* = 0.88, *p* = 0.47, η^2^ = 0.01) or between crime type and language (*F_2,328_* = 0.54, *p* = 0.58, η^2^ = 0.003).

**Punishment ratings**

To explore whether type of crime had an effect on the group differences observed in punishment ratings for accidental harms, we conducted a 3 × 3 x 2 ANOVA, with type of crime (3) as within-subjects factor, and group (3) and language (2) as a between-subjects factor. Across groups and language conditions (*F_2,328_* = 46.63, *p* > 0.00001, η^2^ = 0.23), participants assigned more punishment to death (*p* = 0.0002) and physical harm (*p* = 0.00002) scenarios compared to property damage scenarios. No difference was observed between ratings for death and physical harm (*p* = 0.95). There was also a significant main effect of group (*F_2,163_* = 4.20, *p* = 0.01, η^2^ = 0.04). Judges and attorneys punished accidental harmful actions to a similar degree (*p* = 0.97). However, controls punished accidental transgressions more than judges (*p* = 0.02) and attorneys (*p* = 0.02).

There were no significant interactions between type of crime and group (*F_4,328_* = 0.76, *p* = 0.54, η^2^ = 0.009) or between type of crime and language (*F_2,328_* = 1.27, *p* = 0.28, η^2^ = 0.007).

**Harm severity ratings**

To explore whether crime type had an effect on the group differences observed in harm severity ratings for accidental scenarios, we conducted a 3 × 3 x 2 ANOVA, with crime type (3) as a within-subject factor, and group (3) and language (2) as a between-subjects factor. Across groups and language conditions (*F_2,328_* = 6.84, *p* = 0.001, η^2^ = 0.23), participants assigned higher harm severity ratings to death compared to physical harm (*p* = 0.05) and property damage (*p* = 0.0006) scenarios. No difference was observed between ratings for physical harm and property damage scenarios (*p* = 0.31). A significant main effect of group (*F_2,163_* = 8.87, *p* = 0.0002, η^2^ = 0.09) revealed that judges (*p* = 0.00006) and attorneys (*p* = 0.02) assigned significantly lower severity harm ratings to accidental harmful actions than did controls. Judges and attorneys did not differ in their harm severity ratings for the accidental condition (*p* = 0.15).

There were no significant interactions between crime type and either group (*F_4,328_* = 1.17, *p* = 0.32, η^2^ = 0.01) or language (*F_2,328_* = 2.62, *p* = 0.08, η^2^ = 0.01).

| **Table S1.** Demographic data, global cognitive state, executive functions assessment. | | | | | |
| --- | --- | --- | --- | --- | --- |
|  | | | | | |
|  |  | **Attorneys** | **Controls** | **Judges** |  |
|  |  | **(*N* = 30)** | **(*N* = 27)** | **(*N* = 29)** | ***p*** |
|  |  | **Mean [95%CI]** | **Mean [95%CI]** | **Mean [95%CI]** | **Values** |
| **Demographics** | Age (years) | 35.7 [31.6, 39.8] **^a^** | 42.4 [37.8, 47.0] | 45.86 [42.3, 49.5] | 0.00*** |
|  | Gender (M:F) | 16:14 | 16:11 | 16:13 | 0.90 |
|  | Education (years) | 19.3 [18.5, 20.1] | 19.9 [18.7, 21.1] | 20.6 [19.6, 21.6] | 0.17 |
|  | Experience (years) | 9.8 [6.32, 13.2] | N/A | 17.2 [13.5, 21.0] | 0.00** |
| **Global cognitive functioning and executive functions assessments** | MoCA | 25.3 [24.4, 26.3] | 25.6 [24.5, 26.7] | 26.4 [25.4, 27.3] **^+^** | 0.32 |
|  | IFS total score | 23.9 [23.0, 24.8] | 25.0 [23.9, 26.2] | 24.8 [23.9, 25.7] | 0.21 |
|  | Motor series | 2.8 [2.6, 3.0] | 3.0 [2.9, 3.0] | 2.8 [2.6, 3.0] | 0.38 |
|  | Conflicting instructions | 2.7 [2.4, 3.0] | 2.9 [2.7, 3.1] | 3.0 [3.0, 3.0] | 0.20 |
|  | Go- no go | 2.5 [2.1, 2.9] | 2.1 [1.8, 2.5] | 2.4 [2.2, 2.7] | 0.29 |
|  | Backward digits span | 4.0 [3.6, 4.3] | 4.3 [3.9, 4.8] | 4.3 [3.9, 4.7] | 0.31 |
|  | Verbal working memory | 1.9 [1.8, 2.1] | 1.8 [1.7, 2.0] | 1.9 [1.8, 2.0] | 0.34 |
|  | Spatial working memory | 2.7 [2.4, 3.1] | 2.9 [2.5, 3.4] | 2.7 [2.4, 3.1] | 0.63 |
|  | Abstraction capacity | 2.1 [1.8, 2.4] | 2.5 [2.2, 2.7] | 2.5 [2.3, 2.7] | 0.02 * |
|  | Verbal inhibitory control | 5.1 [4.7, 5.5] | 5.4 [5.1, 5.7] | 5.1 [4.7, 5.5] | 0.41 |
| IFS: INECO Frontal Screening battery | | | | | |
| MoCA: Montreal Cognitive Assessment | | | | | |
| **^a^** Attorneys were significantly younger than controls, and there was a statistical tendency for attorneys to be younger than judges.  N/A Not applicable, | | | | | |
| + One missing observation. | | | | | |
| Significance coding: * p<0.05; **p<0.01; *** p<0.001; **** p<0.0001. | | | | | |

**Supplementary references**

Baez S, Ibanez A, Gleichgerrcht E et al (2014) The utility of IFS (INECO Frontal Screening) for the detection of executive dysfunction in adults with bipolar disorder and ADHD. *Psychiatry Res* *216*: 269-276. doi: 10.1016/j.psychres.2014.01.020

Bruno D, Torralva T, Marenco V et al (2015) Utility of the INECO frontal screening (IFS) in the detection of executive dysfunction in patients with relapsing-remitting multiple sclerosis (RRMS). *Neurol Sci*. doi: 10.1007/s10072-015-2299-6

Clifford GD, Tarassenko L (2005) Quantifying errors in spectral estimates of HRV due to beat replacement and resampling. *IEEE Trans Biomed Eng* *52*: 630-638. doi: 10.1109/TBME.2005.844028

Task Force of the European Society of Cardiology and the North American Society of Pacing and Electrophysiology (1996) Heart rate variability: standards of measurement, physiological interpretation and clinical use. *Circulation* *93*: 1043-1065.

Gleichgerrcht E, Roca M, Manes F et al (2011) Comparing the clinical usefulness of the Institute of Cognitive Neurology (INECO) Frontal Screening (IFS) and the Frontal Assessment Battery (FAB) in frontotemporal dementia. *J Clin Exp Neuropsychol* *33*: 997-1004. doi: 10.1080/13803395.2011.589375

Hejjel L (2004) Suppression of power-line interference by analog notch filtering in the ECG signal for heart rate variability analysis: to do or not to do? *Med Sci Monit* *10*: MT6-13.

Julayanont P, Brousseau M, Chertkow H et al (2014) Montreal Cognitive Assessment Memory Index Score (MoCA-MIS) as a predictor of conversion from mild cognitive impairment to Alzheimer's disease. *J Am Geriatr Soc* *62*: 679-684. doi: 10.1111/jgs.12742

Moreira HS, Lima CF, Vicente SG (2014) Examining Executive Dysfunction with the Institute of Cognitive Neurology (INECO) Frontal Screening (IFS): normative values from a healthy sample and clinical utility in Alzheimer's disease. *J Alzheimers Dis* *42*: 261-273. doi: 10.3233/JAD-132348

Ruha A, Sallinen S, Nissila S (1997) A real-time microprocessor QRS detector system with a 1-ms timing accuracy for the measurement of ambulatory HRV. *IEEE Trans Biomed Eng* *44*: 159-167. doi: 10.1109/10.554762

Torralva T, Roca M, Gleichgerrcht E et al (2009) INECO Frontal Screening (IFS): a brief, sensitive, and specific tool to assess executive functions in dementia. *J Int Neuropsychol Soc* *15*: 777-786. doi: 10.1017/S1355617709990415
